# Trends in Mortality Related to Atrial Fibrillation and Dementia in Older Adults in the United States: A 2000–2020 Analysis

**DOI:** 10.1111/jce.16644

**Published:** 2025-03-24

**Authors:** Muhammad U. Sohail, Ruqiat M. Batool, Muhammad Saad, Saad A. Waqas, Muhammed A. Noushad, Muhammad O. Sohail, Matthew Bates, Raheel Ahmed, David Ripley

**Affiliations:** ^1^ Department of Medicine Dow University of Health Sciences Karachi Pakistan; ^2^ University Hospitals Plymouth NHS Trust Plymouth UK; ^3^ Conemaugh Memorial Medical Center Johnstown Pennsylvania USA; ^4^ James Cook University Hospital Middlesbrough UK; ^5^ National Heart and Lung Institute, Imperial College London London UK; ^6^ Northumbria Hospitals NHS Foundation Trust North Shields UK; ^7^ University of Sunderland Sunderland UK

**Keywords:** atrial fibrillation, CDC WONDER database, dementia, mortality trends, older adults

## Abstract

**Background:**

Atrial fibrillation (AF) and dementia are increasingly prevalent in aging US populations. Their association raises public health concerns, emphasizing the need to understand mortality trends in older adults. This study examines AF and dementia‐related mortality trends from 2000 to 2020.

**Methods:**

Using the CDC WONDER Multiple Cause of Death database, we analyzed death certificates for individuals aged 65 and older, reporting age‐adjusted mortality rates (AAMRs) per 100 000 persons. Trends were assessed through annual percent change (APC) analysis via Joinpoint regression, with stratifications by sex, race/ethnicity, urbanization, and Census regions.

**Results:**

A total of 400 103 AF and dementia‐related deaths were recorded between 2000 and 2020. The AAMR increased markedly from 25.4 in 2000 to 70.4 in 2020. The overall AAMR showed a steady increase from 2000 to 2018 (APC: +4.2%; 95% CI: 2.5–5.5), with a sharper rise from 2018 to 2020 (APC: +9.5%; 95% CI: 4.5–12.2; *p* < 0.001). Mortality rates were comparable between men (AAMR: 44.4) and women (AAMR: 43.9). NH White individuals exhibited the highest AAMR (47.0), followed by NH Black (26.6), Hispanic (23.1), and NH Asian/Pacific Islander (18.0) populations. Nonmetropolitan areas had higher AAMRs (48.1) compared to metropolitan areas (43.5). Regionally, the Western US recorded the highest AAMR at 48.2, while state‐level disparities showed a nearly threefold difference between the top 90th and bottom 10th percentiles.

**Conclusion:**

Rising AF and dementia‐related mortality rates among older adults highlight a need for targeted screening and intervention, particularly for high‐risk demographics and underserved regions.

## Graphical abstract

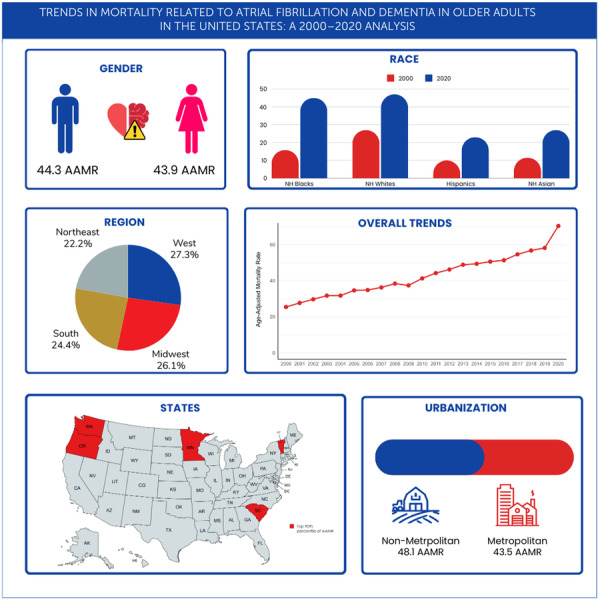

## 1 Introduction

Atrial fibrillation (AF) is the most prevalent sustained arrhythmia globally [1], with its incidence and prevalence expected to rise dramatically in the coming decades. Estimates indicate that by 2050, AF will affect up to 15.9 million individuals in the United States alone, driven largely by the aging population [2, 3, 4]. AF predominantly impacts individuals over 65 [5], with nearly 70% of cases occurring in those aged 65–85 [6], and approximately 10% of people older than 80 affected [7]. Similarly, dementia poses a growing public health challenge worldwide, with projections suggesting that the incidence will double with every 5.9‐year increase in age. By 2030, over 75 million people worldwide will be living with dementia, and this number is expected to surpass 135 million by 2050 [8]. Together, AF and dementia represent a significant health and economic burden, especially in aging populations.

There is a well‐documented association between AF and an elevated risk of cognitive impairment and dementia [9, 10]. A large meta‐analysis reported that AF is associated with a 39% increase in the risk of cognitive impairment among the general population [11]. Increasing research efforts are shedding light on the complex interplay between AF and dementia, including shared pathophysiological mechanisms, therapeutic management strategies, and outcomes [12, 13]. Studies have shown that mortality rates related to both AF [14] and dementia [15] are rising, yet the specific trends in AF and Dementia related mortality among older adults in the United States remain unclear. This study aims to examine mortality trends associated with AF and dementia among older adults from 2000 to 2020. Utilizing data from the US Centers for Disease Control and Prevention's (CDC) Wide‐Ranging Online Data for Epidemiologic Research (WONDER) system, our analysis focuses on variations across sex, race, urbanization, and geographic regions to provide a comprehensive understanding of these intersecting health concerns.

## 2 Materials and Methods

### 2.1 Study Setting and Population

This descriptive study examined mortality data derived from death certificates using the CDC WONDER database. The primary objective was to analyze trends in mortality rates related to AF and dementia among individuals aged 65 and older over the period from 2000 to 2020.

We focused on records within the Multiple Cause of Death (MCD) Public Use data set, which encompasses mortality causes across all 50 US states and the District of Columbia. This data set is widely utilized in mortality trend research. To identify AF, we used ICD‐10 code I48, and for dementia, we applied ICD‐10 codes F01, F03, and G30 as contributing causes of death. These codes have been validated in prior studies using the CDC WONDER database to accurately capture AF and dementia‐related mortalities [14, 15]. Additionally, we conducted a sensitivity analysis to examine deaths where dementia was specifically listed as a contributing cause of death.

As this study utilized deidentified, publicly accessible data from a government database, it did not require institutional review board (IRB) approval. The study adhered to the Strengthening the Reporting of Observational Studies in Epidemiology (STROBE) guidelines.

### 2.2 Data Abstraction

Data were stratified by demographic and geographic characteristics, including gender, race/ethnicity, urbanization level, census region, and state of residence. Racial and ethnic classifications were defined as Hispanic or Latino, Non‐Hispanic (NH) White, and NH Black/African American, using categories from death certificate data commonly applied in previous WONDER database analyses [16].

Urban‐rural status was assigned according to the National Center for Health Statistics (NCHS) Urban‐Rural Classification Scheme, categorizing counties as urban (large metropolitan areas with populations over 1 million and medium/small metropolitan areas with populations between 50 000 and 999 999) or rural (populations under 50 000), as per the 2013 US Census [17]. Geographic regions were classified into Northeast, Midwest, South, and West, following US Census Bureau guidelines [18].

### 2.3 Statistical Analysis

To assess national trends in AF and dementia‐related mortalities, we calculated crude mortality rates (CMRs) and age‐adjusted mortality rates (AAMRs) per 100 000 individuals, standardizing to the year 2000 United States population as the baseline [18]. CMRs were obtained by dividing AF and dementia‐related deaths by the United States population of each year, while AAMRs were adjusted to the 2000 baseline.

To evaluate changes in mortality rates over time, we employed the Joinpoint Regression Program (Version 5.2.0, National Cancer Institute) [19]. This program uses log‐linear regression models to estimate the annual percent change (APC) in AAMR along with a 95% confidence interval (CI). APCs were classified as increasing or decreasing based on their deviation from a null hypothesis of zero change, with statistical significance set at *p* < 0.05, using a two‐tailed *t*‐test. Additionally, a parallelism test was conducted to determine if trends across different groups were statistically similar or distinct; a significant *p*‐value in this test indicated a meaningful difference between average annual percent change (AAPC) trends across groups [20].

## 3 Results

A total of 400 103 AF and dementia‐related deaths among older adults (aged ≥ 65 years) occurred between 2000 and 2020. Of these, 39 105 deaths had AF as the underlying cause of death with dementia as a contributing cause, while 156 877 deaths had dementia as the underlying cause of death with AF as a contributing cause. Additionally, 204 121 deaths were recorded where neither AF nor dementia was listed as the underlying cause of death. Information on the location of death was available for 376 857 deaths. Of these, 55.1% occurred in nursing homes/long‐term care facilities, 21.9% in medical facilities, 18.0% at the decedent's home, and 4.9% in hospices (Supporting Information S1: Table S1).

### 3.1 Annual Trends for AF and Dementia‐Related Mortality

**Figure 1:**
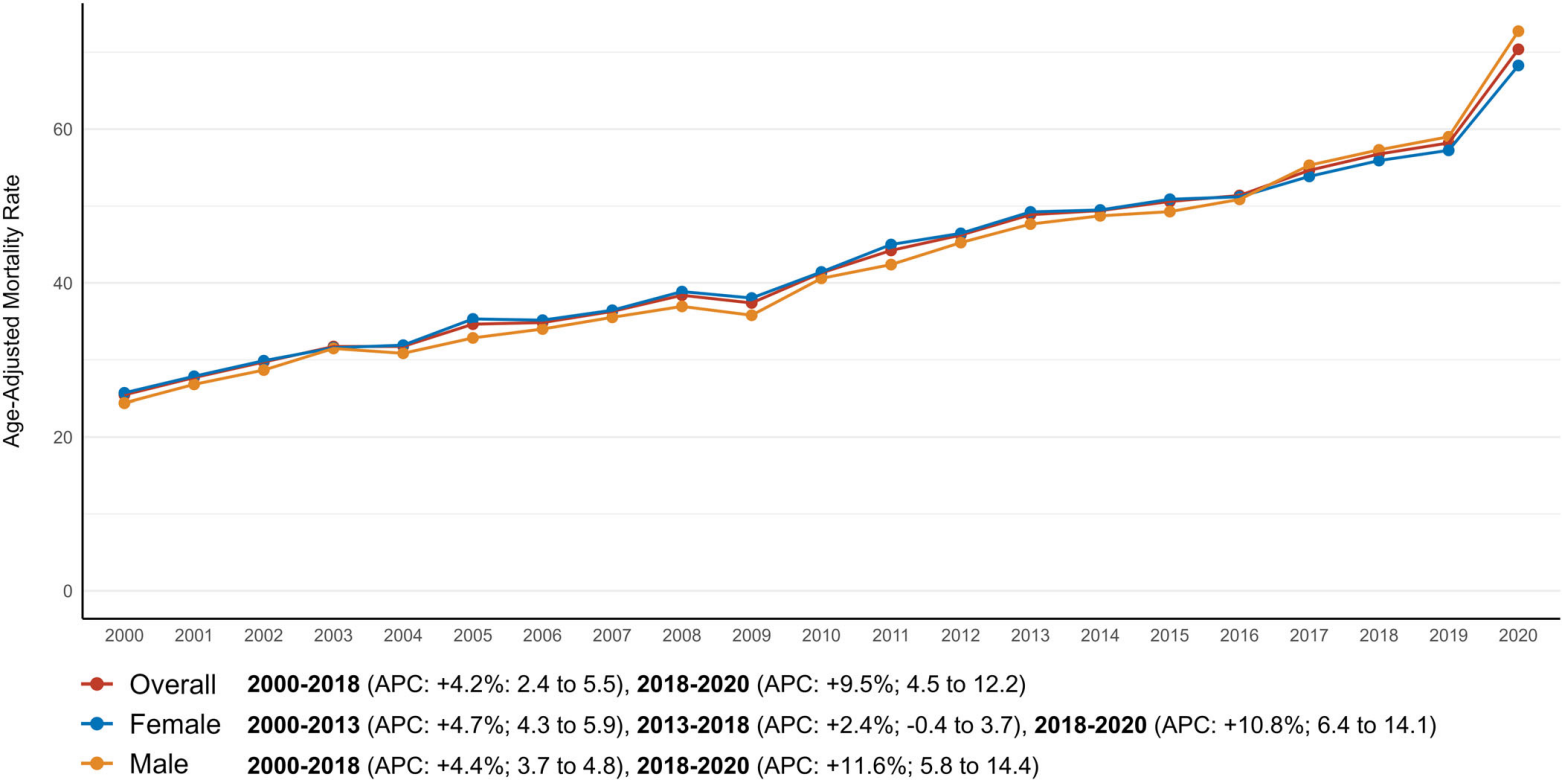
Overall and sex‐stratified atrial fibrillation and dementia‐related AAMRs per 100 000 in older adults in the United States from 2000 to 2020.

The AAMR for AF and dementia‐related deaths in older adults increased markedly from 25.4 in 2000 to 70.4 in 2020. The overall AAMR rose consistently from 2000 to 2018 (APC: +4.2%; 95% CI: 2.4–5.5), followed by an accelerated increase from 2018 to 2020 (APC: +9.5%; 95% CI: 4.5–12.2), highlighting a substantial rise in recent years (Figure 1, Supporting Information S1: Table S2). When compared to mortalities related to dementia, trends for AF and dementia‐related mortalities turned out to be significantly different (*p* < 0.001) (Table 1, Supporting Information S1: Figure S1).

**Table 1:** Age‐adjusted mortality rate trend in US subjects with atrial fibrillation and dementia, 2000–2020.

	Deaths	AAMR 2000 (95% CI)	AAMR 2020 (95% CI)	AAPC (95% CI)	*p* (for parallelism)
Dementia in total versus AF population					*p* < 0.001
Dementia in total population	6 367 889	581.7 (579.2–584.2)	855.1 (852.6–857.7)	+1.5% (1.1–1.9)	
Dementia in AF population	400 103	25.4 (24.9–26.0)	70.4 (69.7–71.1)	+4.7% (4.2–5.0)	
Gender					*p* < 0.001
Men	143 515	24.4 (23.5–25.3)	72.7 (71.6–73.9)	+5.1% (4.6–5.4)	
Women	256 588	25.7 (25.1–26.4)	68.3 (67.4–69.2)	+4.7% (4.4–5.0)	
Non‐Hispanic Race					
NH Asian/Pacific Islander	5336	11.5 (9.0–14.6)	27.5 (25.4–29.5)	+4.8% (4.1–6.9)	NH Asian/Pacific Islander versus NH White: *p* = 0.75
NH Black/African American	18 685	15.8 (14.3–17.3)	45.2 (43.3–47.2)	+5.3% (4.8–6.1)	NH Black versus NH Asian/Pacific Islander: *p* = 0.33
NH White	375 103	26.6 (26.1–27.2)	75.6 (74.8–76.4)	+4.9% (4.3–5.2)	NH White versus NH Black individuals: *p* = 0.46
Hispanic	12 154	9.6 (7.9–11.3)	39.7 (37.8–41.7)	+6.8% (6.3–7.8)	Hispanic versus NH White: *p* < 0.001 Hispanic versus NH Black: *p* < 0.001 Hispanic versus NH Asian/Pacific Islander: *p* < 0.001
Urbanization					*p* < 0.001
Metropolitan	322 977	25.5 (24.9–26.1)	67.6 (66.9–68.4)	+4.7% (4.3–5.2)	
Nonmetropolitan	77 126	25.3 (24.1–26.5)	84.0 (82.0–85.9)	+5.6% (4.9–6.2)	
Census region					
Northeast	34 872	24.5 (23.4–25.6)	63.9 (62.4–65.5)	+4.4% (4.0–4.9)	—
Midwest	46 207	26.7 (25.6–27.8)	78.9 (77.2–80.5)	+5.1% (4.4–5.5)	—
South	64 758	23.3 (22.4–24.2)	69.7 (68.5–70.9)	+5.2% (4.9–5.6)	—
West	51 616	28.9 (27.6–30.2)	68.4 (66.9–69.9)	+4.2% (3.6–4.8)	—

### 3.2 AF and Dementia‐Related Mortality Trends Stratified by Gender

Trends in mortalities differed by gender (*p* < 0.001). The overall AAMR was 44.4 (95% CI: 44.2–44.6) for older men and 43.9 (95% CI: 43.8 –44.1) for older women (Table 1). For older men, the AAMR increased from 24.4 in 2000 to 72.7 in 2020, showing a steady rise from 2000 to 2018 (APC: +4.4%; 95% CI: 3.7–4.8) and a sharper increase from 2018 to 2020 (APC: +11.6%; 95% CI: 5.8–14.4). For older women, the AAMR rose from 25.7 in 2000 to 68.28 in 2020, with an initial rise from 2000 to 2013 (APC: +4.7%; 95% CI: 4.3–5.9), a slower increase from 2013 to 2018 (APC: +2.4%; 95% CI: –0.4 to 3.7), and an accelerated rise from 2018 to 2020 (APC: +10.8%; 95% CI: 6.4–14.1) (Figure 1, Supporting Information S1: Table S2).

### 3.3 AF and Dementia‐Related Mortality Trends Stratified by Race

The overall AAMR was highest among NH White adults (47.0; 95% CI: 46.8–47.1), followed by NH Black/African American adults (26.6; 95% CI: 26.2–27.0), Hispanic adults (23.1; 95% CI: 22.7–23.5), and NH Asian/Pacific Islander adults (18.0; 95% CI: 17.5–18.5) (Table 1).

**Figure 2:**
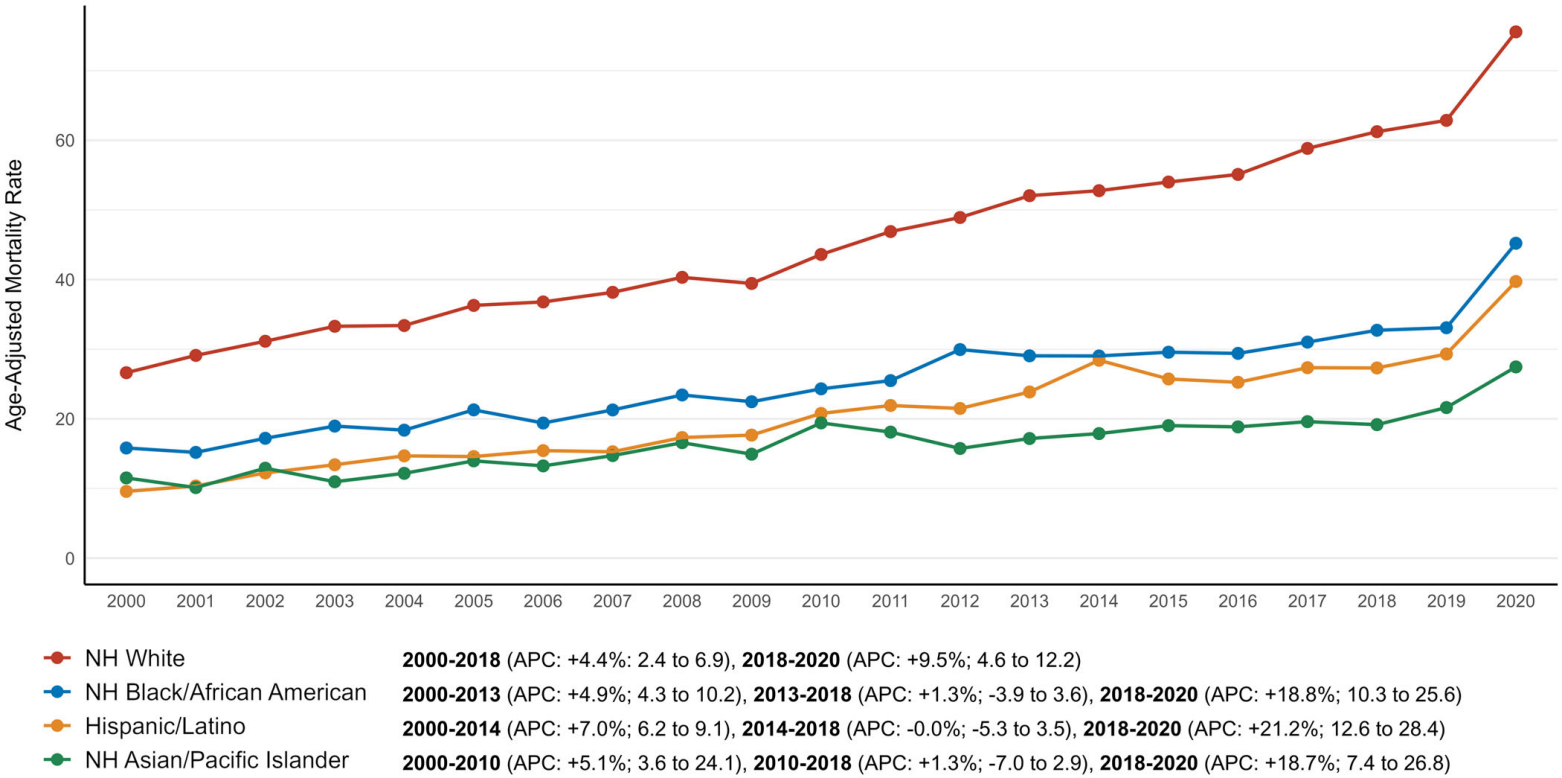
Atrial fibrillation and dementia‐related AAMRs per 100 000 stratified by race in older adults in the United States from 2000 to 2020.

NH White adults experienced a steady increase from 2000 to 2018 (APC: +4.4%; 95% CI: 2.4–6.9), with a sharper rise from 2018 to 2020 (APC: +9.5%; 95% CI: 4.6–12.2). Among NH Black/African American adults, the AAMR rose from 2000 to 2013 (APC: +4.9%; 95% CI: 4.2–10.2), slowed from 2013 to 2018, and then rose notably from 2018 to 2020 (APC: +18.8%; 95% CI: 10.3–25.6). Hispanic adults showed an increase from 2000 to 2014 (APC: +7.0%; 95% CI: 6.2–9.1), plateaued from 2014 to 2018 (APC: –0.0%; 95% CI: –5.3 to 3.5), and sharply increased from 2018 to 2020 (APC: +21.2%; 95% CI: 12.6–28.4). NH Asian/Pacific Islander adults had the lowest rates, with an initial rise from 2000 to 2010 (APC: +5.1%; 95% CI: 3.6–24.1), a minimal rise from 2010 to 2018 (APC: 1.3%; 95% CI: –7.0 to 2.9), and an increase from 2018 to 2020 (APC: +18.7%; 95% CI: 7.4–26.8) (Figure 2, Supporting Information S1: Table S2).

The Hispanic population exhibited a significantly larger rise compared to other racial groups (*p* < 0.001 vs. NH Asian/Pacific Islander, NH Black/African American, and NH White). In contrast, all other comparisons showed nonsignificant results in the test for parallelism (*p* > 0.3) (Table 1).

### 3.4 AF and Dementia‐Related Mortality Trends Stratified by Geography

**Figure 3:**
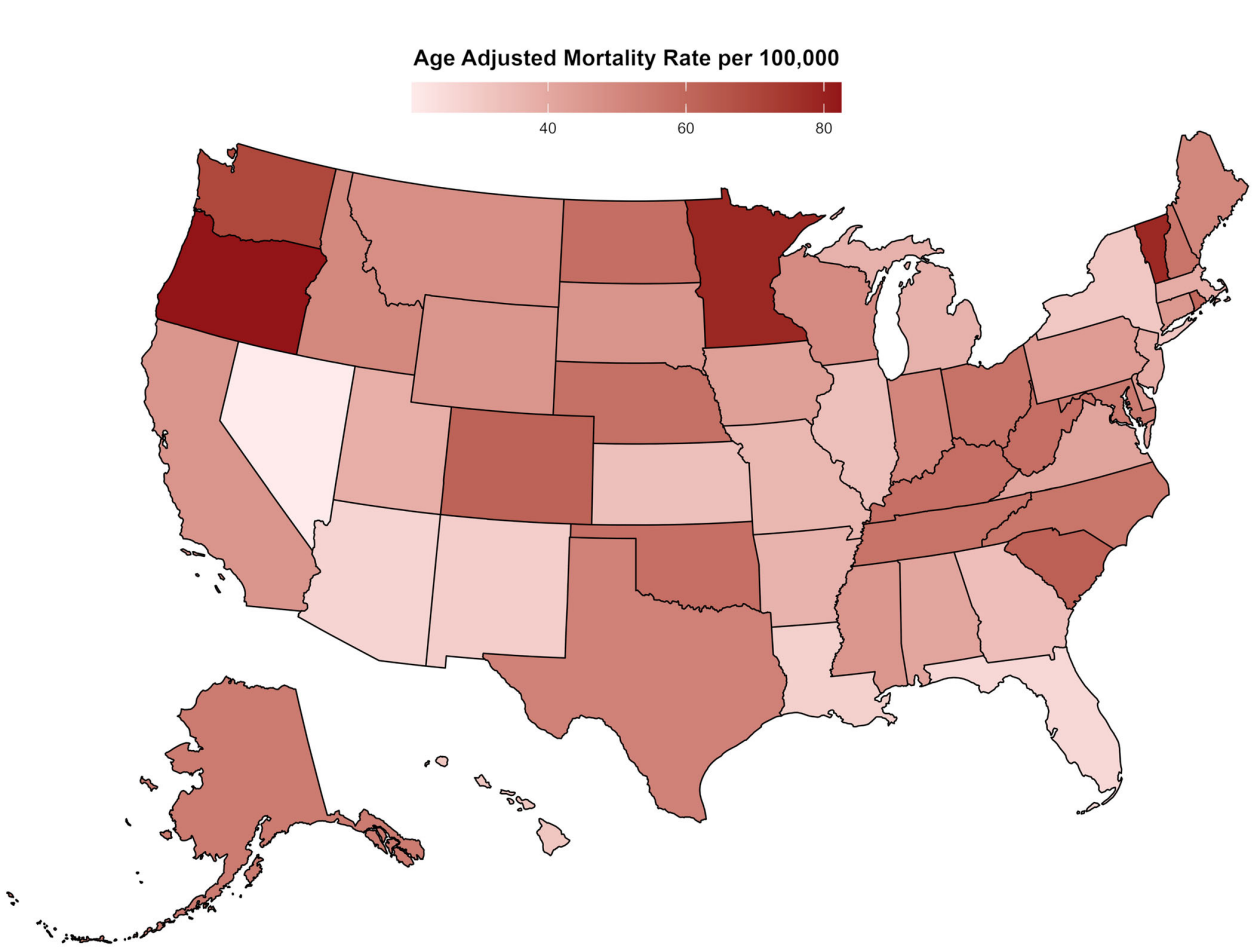
Atrial fibrillation and dementia‐related AAMRs per 100 000 stratified by state in older adults in the United States from 2000 to 2020.

A significant difference in AAMRs was observed in different states, with the AAMRs ranging from 20.3 (95% CI: 19.1–21.5) in Nevada to 82.5 (95% CI: 80.9–84.1) in Oregon. States that fell into the top 90th percentile were Oregon, Minnesota, Vermont, Washington, and South Carolina, which had almost triple the AAMRs compared with states that fell into the lower 10th percentile, namely, Nevada, Florida, District of Columbia, Arizona, and Louisiana (Figure 3, Supporting Information S1: Table S3). Heat maps were generated at 5‐year intervals from 2000 to 2020 to visualize trends in AF and dementia‐related mortality across US states (Supporting Information S1: Figures S2–S5). On average, over the course of the study period, the highest mortality was observed in the Western (AAMR: 48.2; 95% CI: 47.9–48.5), followed by the Midwestern (AAMR: 46.5; 95% CI: 46.2–46.7), Southern (AAMR: 43.4; 95% CI: 43.1–43.6), and Northeast (AAMR: 39.3; 95% CI: 39.0–39.6) regions (Table 1, Supporting Information S1: Figure S6, Supporting Information S1: Table S4).

**Figure 4:**
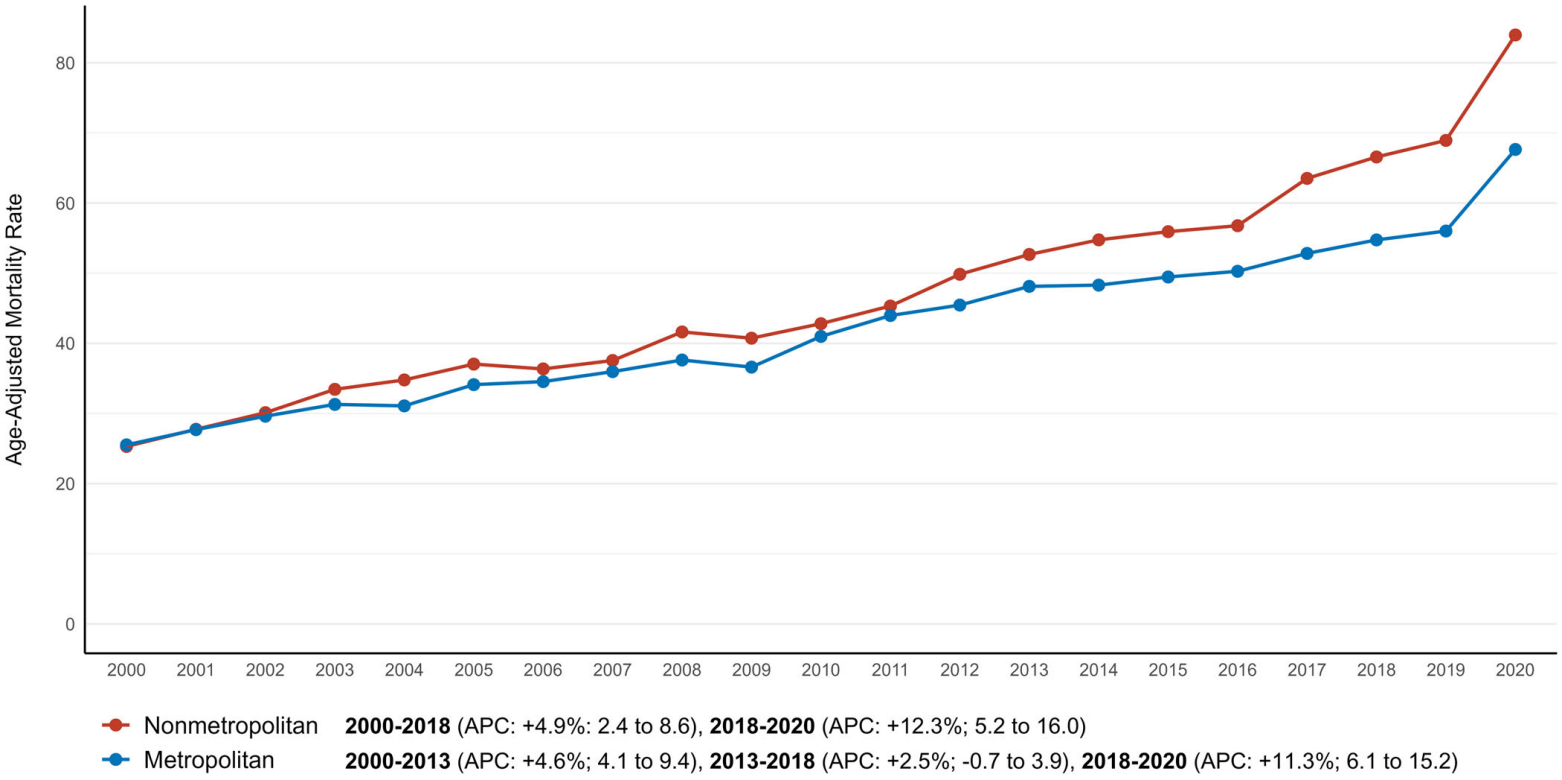
Atrial fibrillation and dementia‐related AAMRs per 100 000 stratified by urbanization in older adults in the United States from 2000 to 2020.

Nonmetropolitan areas exhibited a higher AAMR than metropolitan areas. (*p* < 0.001) (Table 1). The AAMR was 48.1 (95% CI: 47.7–48.4) for nonmetropolitan areas and 43.5 (95% CI: 43.3–43.6) for metropolitan areas. In nonmetropolitan areas, AAMRs rose consistently at an APC of +4.9% (95% CI: 2.4–8.6) from 2000 to 2018, accelerating to +12.3% (95% CI: 5.2–16.0) between 2018 and 2020. In metropolitan areas, AAMRs increased steadily, with an APC of +4.6% (95% CI: 4.1–9.4) from 2000 to 2013, followed by a slower rise of +2.5% (95% CI: –0.68 to 3.9) between 2013 and 2018, and a sharp increase to +11.3% (95% CI: 6.1–15.2) from 2018 to 2020 (Figure 4, Supporting Information S1: Table S5).

## 4 Discussion

This 21‐year analysis of CDC WONDER data analyzed mortality trends in people with concomitant AF and Dementia. Our findings indicate a consistent increase in mortality from 2000 to 2018, followed by a steeper increase till 2020, underscoring a concerning rise across all demographics. Mortality rates were markedly higher among NH Whites compared to NH African Americans, with men experiencing slightly elevated rates compared to women. Notable geographic disparities were also identified: nonmetropolitan areas exhibited higher mortality rates than metropolitan areas, and mortality was highest in the Western region. Additionally, states within the top 90th percentile of AAMRs demonstrated nearly triple the mortality rates than states in the bottom 10th percentile. These results have important public health policy implications.

The association between AF and dementia is well‐supported in current literature, reinforcing our findings. A large US cohort study of 37 025 older adults found that AF independently correlates with an increased risk of developing dementia, as well as elevated mortality rates among those with both AF and dementia compared to those with dementia alone [21]. Furthermore, In a study by Zhang et al., which followed 433 746 participants over a median period of 12.6 years, individuals with AF exhibited a substantially elevated risk of developing dementia compared to those without AF [22]. Several mechanisms may contribute to this, including cerebral hypoperfusion from AF [23], leading to amyloid‐beta deposition, silent cerebral infarcts [24], microinfarcts, and cerebral hemorrhages [25], all of which are linked to cognitive decline. AF also elevates inflammatory markers [26], which are associated with dementia progression, and increases the risk of ischemic stroke, a known dementia risk factor [27, 28].

We observed a consistent upward trend in mortality, which aligns with findings from Kouki et al., who documented a rise in AF and dementia‐related deaths during the study period from 2011 to 2017 [29]. This trend is driven by the aging US population [30], which faces growing cardiometabolic risks like hypertension, diabetes, and obesity—conditions linked to higher all‐cause and cardiovascular mortality in AF‐dementia patients [31]. AF further increases stroke risk, while comorbidities such as heart failure and chronic kidney disease worsen outcomes [31]. Despite rising dementia awareness, underdiagnosis remains common, often occurring at advanced stages, leading to poor prognosis [32]. Mobility impairments and limited public recognition of AF (only 48%) further delay timely diagnosis and management, contributing to higher mortality [33, 34]. Advancements in neuroimaging (MRI, CT, PET, and SPECT) and biomarker detection have improved dementia diagnosis, potentially increasing reported deaths [35, 36]. The sharp rise from 2019 to 2020 may reflect the COVID‐19 pandemic's impact on healthcare and disease management. Patients with AF and Dementia are typically older with multiple comorbidities, making them more vulnerable to severe COVID‐19 complications. Additionally, disruptions in healthcare access and delayed routine care worsened disease management, while increased medical scrutiny during the pandemic may have led to more accurate reporting and diagnosis on death certificates.

Our findings indicate similar mortality trends in patients with AF and Dementia for both genders, with men exhibiting slightly higher mortality rates. Previous studies suggest older women may have a higher incidence of AF and dementia due to the loss of estrogen's protective effect [37], along with a greater prevalence of hypertension and diabetes [38] and less frequent use of DOACs [39], which provide protective effects on cognitive function and a reduced risk of AF‐related dementia [40]. Our findings, indicating a slightly higher AAMR for men, may be attributed to the increased fatality of AF when combined with other cardiovascular conditions, which are more prevalent in older men [41]. This observation aligns with Ptack et al.'s prediction of higher mortality risks in men with coexisting AF and dementia, highlighting a possible cumulative effect on fatality rates in this demographic [42]. Given these nuances, further research is essential to clarify gender‐based differences in AF‐related mortality.

Our results also highlight important racial and ethnic disparities in patients. Throughout the study period, mortality trends remained highest in White individuals compared to their Black counterparts. A meta‐analysis [43] incorporating data from the ARIC and CHS studies indicated that a 10% increase in European ancestry was associated with a 13% higher risk of AF [43]. The factors identified to explain racial disparities in AF may also help clarify disparities in AF‐dementia comorbidity, given the established link between AF and dementia. This observation aligns with the widely studied phenomenon known as the “AF race paradox,” which describes the lower prevalence of AF in Black individuals compared to Whites despite a higher prevalence of modifiable risk factors such as obesity, hypertension, and diabetes among Black populations [44].

Genetic factors play a key role in these disparities. Variations at the PITX2 locus are linked to AF, and certain SNPs in this region are associated with dementia in Caucasians [45]. A protective minor allele of a PITX2‐related SNP is more common in Black individuals, reducing their AF risk by 11.4%–31.7% [46]. This may partly explain why AF incidence and mortality are higher in Whites. Detection differences also contribute. White men tend to have larger left atria, making AF easier to detect, while Black individuals may have more undiagnosed cases due to studies missing paroxysmal or silent AF episodes [47, 48]. Lower awareness of AF and dementia, reduced health literacy, limited healthcare access, and provider bias further worsen disparities [49, 50, 51].

Significant geographical disparities in AF dementia‐related mortality rates reveal unique challenges faced by rural populations. First, rural residents may experience a higher risk for the development of AF due to risk factors such as smoking, hypertension, diabetes, and obesity, compared to urban counterparts, which collectively increase their susceptibility to developing dementia [52]. Second, limited awareness and self‐management pose challenges, as many rural individuals have less knowledge of AF and its implications [53]. This limited awareness is compounded by restricted access to specialty care, high medication costs, and a shortage of emergency transport [54]. The “invisibility” of AF, wherein symptoms are not readily apparent to others, adds a layer of isolation, making rural patients more reliant on interpersonal support and technology for self‐care—strategies that may not suffice for optimal disease management [54]. Third, rural regions experience substantial shortages of healthcare providers, particularly specialists such as electrophysiologists, which often leads patients to rely on urgent care rather than specialized treatment [55]. As a result, rural patients are less likely to receive DOACs, further impacting their quality of care and outcome [56]. Finally, financial barriers add another layer of complexity, as rural populations are more likely to rely on Medicare and Medicaid, limiting their access to copayment discounts and other forms of financial assistance available to commercially insured patients [57]. Being underinsured or reliant on public insurance can significantly hinder access to necessary medications, specialized consultations, and continuous care, thereby intensifying health disparities [58]. AF‐related dementia mortality patterns vary geographically, with notable differences between the Southern and Western regions. While the South has high AF prevalence but lower mortality, the West shows the highest AF‐related stroke mortality, suggesting potential parallels in AF‐dementia trends [59]. Major geographic variation in dementia mortality could be due to differences in disease prevalence, influenced by incidence and duration. Higher reported mortality in some states may stem from better diagnostic practices, higher awareness, and access to specialist care, leading to more accurate reporting [60]. Further investigation at the county level is needed to better understand localized risk factors and disparities in AF‐dementia outcomes.

Evidence suggests that AF patients on oral anticoagulants face a significantly lower risk of developing dementia compared to those who do not receive anticoagulation therapy [61]. This finding underscores the critical role of consistent anticoagulant use in effective AF management. Additionally, rhythm‐control strategies, such as catheter ablation and cardioversion, not only stabilize heart rhythm but may also enhance cerebral perfusion, potentially preventing the onset of dementia [62]. Addressing modifiable risk factors through lifestyle modifications is equally crucial. Antihypertensive therapies [63] and lipid‐lowering agents like atorvastatin and ezetimibe have shown efficacy in preserving neurocognitive function in older AF patients [64]. Encouraging regular physical activity, maintaining a balanced diet, and engaging in cognitive stimulation can further support brain health and help mitigate cognitive decline associated with AF [65]. Moreover, expanding the role of the CHA2DS2‐VASc score in early risk stratification can aid in identifying high‐risk patients [66], facilitating more targeted and effective care. Legislative initiatives aimed at increasing funding for emergency medical services in rural areas are also essential, as they can improve access to advanced AF management and reduce disparities in care [54]. Lastly, ensuring consistent adherence to established clinical guidelines for anticoagulation therapy, ventricular rate control, and effective management of comorbid cardiac conditions across all states and regions could help reduce geographic disparities in the treatment and outcomes of AF and dementia [67, 68].

Patients with high CHA2DS2‐VASc scores should be closely monitored for early cognitive decline [69]. Integrated screening for AF and mild cognitive impairment can enable timely intervention [70]. Future research should focus on developing accessible biomarkers for vascular dementia, as MRI has limitations in predicting early progression [71]. Additionally, more trials should evaluate the effectiveness of various anticoagulation and rhythm‐control strategies, ensuring diverse racial and ethnic representation to understand differential outcomes [65, 69, 72]. Studying the cognitive effects of anticoagulants and potential anti‐inflammatory benefits of statins could help mitigate AF‐related cognitive decline [73]. Additionally, increasing public awareness regarding early detection and prevention strategies will empower patients to seek timely care [13]. This can be done through utilizing consumer‐grade devices, such as smartphone‐paired monitors and smartwatches, which may present a valuable opportunity due to their high sensitivity and specificity for detecting AF [13, 74]. Enhancing these devices with user‐friendly interfaces and longer battery life could improve long‐term monitoring for older adults [75].

This study has several limitations. First, reliance on ICD‐10 codes from death certificates may lead to misclassification of AF and dementia, impacting mortality trends. Second, the CDC WONDER database lacks clinical detail on patients' cardiovascular risk profiles, comorbidities, and disease severity, as well as socioeconomic variables like income, education, and insurance, limiting insight into disparities in care access and outcomes. Third, advancements in medical treatments over the 2000–2020 period are not accounted for, potentially influencing trends. Lastly, the data's cross‐sectional, aggregate nature restricts analysis of individual‐level, longitudinal relationships between AF, dementia, and mortality.

## 5 Conclusion

This study underscores the rising trends in AF‐ and dementia‐related mortality in the United States from 2000 to 2020. AAMRs were similar between men and women, with the highest mortality observed among non‐Hispanic whites. Mortality rates were notably elevated in nonmetropolitan areas and in the Western US region. Our findings highlight the potential of early anticoagulation therapy to reduce cognitive decline in AF patients and the importance of early detection and screening of AF through consumer‐grade technologies, such as smartwatches and smartphones, to help address the increasing burden of mortality.

## Ethics Statement

The authors have nothing to report.

## Conflicts of Interest

The authors declare no conflicts of interest.

## Supporting information

Supporting information.

## Data Availability

The data that support the findings of this study are available in National Center for Health Statistics at https://wonder.cdc.gov/mcd.html. These data were derived from the following resources available in the public domain: CDC WONDER, https://wonder.cdc.gov/.
