# Supplementary material for: Trends in Mortality Related to Atrial Fibrillation and Dementia in Older Adults in the United States: A 2000–2020 Analysis
Source: J Cardiovasc Electrophysiol. 2025 Mar 24;36(6):1234–43. doi: 10.1111/jce.16644 (PMC12160669; doi:10.1111/jce.16644)
Supplement: Supplementary file 1 — Supporting information. [file JCE-36-1234-s001.docx]

| **Place of Death** | **Deaths** |
| --- | --- |
| Medical Facility - Inpatient | 74073 |
| Medical Facility - Outpatient or ER | 7949 |
| Medical Facility - Dead on Arrival | 595 |
| Medical Facility - Status unknown | 75 |
| Decedent's home | 68003 |
| Hospice facility | 18469 |
| Nursing home/long term care | 207703 |
| Other | 22445 |
| Place of death unknown | 791 |
| Total | 400103 |

**Supplementary Table 1.** AF and dementia-related mortality among older adults stratified by place of death in the United States from 2000 to 2020.

| **Year** | **Overall** | **Female** | **Male** | **NH Asian** | **NH Black** | **NH White** | **Hispanic** |
| --- | --- | --- | --- | --- | --- | --- | --- |
| 2000 | 25.44 (24.91-25.98) | 25.7 (25.05-26.35) | 24.39 (23.48-25.31) | 11.52 (8.98-14.55) | 15.82 (14.29-17.34) | 26.63 (26.06-27.2) | 9.59 (7.89-11.28) |
| 2001 | 27.68 (27.13-28.23) | 27.85 (27.17-28.52) | 26.8 (25.85-27.75) | 10.13 (7.85-12.87) | 15.19 (13.7-16.68) | 29.11 (28.52-29.71) | 10.36 (8.65-12.08) |
| 2002 | 29.71 (29.15-30.28) | 29.88 (29.19-30.57) | 28.66 (27.69-29.63) | 12.92 (10.39-15.88) | 17.21 (15.62-18.79) | 31.14 (30.53-31.75) | 12.24 (10.41-14.07) |
| 2003 | 31.72 (31.14-32.3) | 31.56 (30.85-32.27) | 31.47 (30.46-32.47) | 10.97 (8.72-13.62) | 18.96 (17.3-20.61) | 33.29 (32.66-33.91) | 13.41 (11.54-15.27) |
| 2004 | 31.74 (31.16-32.31) | 31.9 (31.19-32.61) | 30.84 (29.86-31.82) | 12.18 (9.78-14.58) | 18.38 (16.76-20.01) | 33.4 (32.78-34.02) | 14.69 (12.78-16.59) |
| 2005 | 34.63 (34.03-35.22) | 35.31 (34.57-36.05) | 32.84 (31.84-33.83) | 13.97 (11.51-16.42) | 21.29 (19.58-23.01) | 36.29 (35.65-36.93) | 14.59 (12.75-16.43) |
| 2006 | 34.84 (34.25-35.43) | 35.15 (34.42-35.88) | 34 (33.01-35) | 13.25 (10.95-15.55) | 19.4 (17.78-21.02) | 36.79 (36.15-37.43) | 15.44 (13.61-17.27) |
| 2007 | 36.29 (35.69-36.88) | 36.44 (35.7-37.18) | 35.52 (34.52-36.52) | 14.73 (12.37-17.1) | 21.28 (19.6-22.95) | 38.17 (37.53-38.81) | 15.28 (13.51-17.04) |
| 2008 | 38.39 (37.79-39) | 38.87 (38.12-39.63) | 36.94 (35.94-37.94) | 16.58 (14.17-19) | 23.43 (21.7-25.16) | 40.32 (39.67-40.97) | 17.32 (15.5-19.14) |
| 2009 | 37.39 (36.8-37.98) | 38.03 (37.29-38.78) | 35.79 (34.82-36.76) | 14.93 (12.71-17.14) | 22.46 (20.79-24.13) | 39.44 (38.79-40.08) | 17.67 (15.89-19.45) |
| 2010 | 41.3 (40.69-41.92) | 41.42 (40.65-42.19) | 40.59 (39.57-41.61) | 19.42 (16.95-21.89) | 24.31 (22.59-26.02) | 43.6 (42.93-44.26) | 20.78 (18.89-22.66) |
| 2011 | 44.22 (43.59-44.84) | 44.99 (44.2-45.78) | 42.38 (41.36-43.4) | 18.1 (15.85-20.36) | 25.49 (23.76-27.21) | 46.89 (46.2-47.57) | 21.92 (20.07-23.76) |
| 2012 | 46.22 (45.59-46.86) | 46.44 (45.64-47.24) | 45.24 (44.21-46.28) | 15.75 (13.72-17.78) | 29.95 (28.12-31.79) | 48.91 (48.22-49.6) | 21.49 (19.72-23.25) |
| 2013 | 48.87 (48.23-49.51) | 49.23 (48.42-50.05) | 47.65 (46.6-48.7) | 17.18 (15.16-19.2) | 29.05 (27.28-30.82) | 52.04 (51.33-52.75) | 23.86 (22.06-25.65) |
| 2014 | 49.4 (48.76-50.04) | 49.48 (48.67-50.29) | 48.72 (47.68-49.76) | 17.89 (15.9-19.88) | 29.03 (27.28-30.77) | 52.76 (52.05-53.46) | 28.42 (26.53-30.32) |
| 2015 | 50.59 (49.95-51.23) | 50.88 (50.07-51.7) | 49.27 (48.23-50.3) | 19.03 (17.07-21) | 29.57 (27.84-31.29) | 54 (53.29-54.71) | 25.73 (23.99-27.47) |
| 2016 | 51.37 (50.73-52.02) | 51.19 (50.38-52) | 50.86 (49.83-51.9) | 18.85 (16.95-20.75) | 29.4 (27.7-31.09) | 55.09 (54.38-55.8) | 25.25 (23.57-26.92) |
| 2017 | 54.64 (53.99-55.3) | 53.85 (53.03-54.68) | 55.3 (54.23-56.37) | 19.6 (17.73-21.48) | 31.02 (29.31-32.73) | 58.83 (58.1-59.56) | 27.35 (25.65-29.04) |
| 2018 | 56.76 (56.11-57.42) | 55.91 (55.08-56.74) | 57.3 (56.23-58.37) | 19.17 (17.38-20.95) | 32.72 (30.99-34.44) | 61.23 (60.49-61.96) | 27.31 (25.66-28.97) |
| 2019 | 58.2 (57.54-58.86) | 57.24 (56.4-58.07) | 59.01 (57.94-60.09) | 21.62 (19.77-23.47) | 33.08 (31.37-34.79) | 62.85 (62.11-63.59) | 29.31 (27.62-31) |
| 2020 | 70.38 (69.66-71.1) | 68.28 (67.37-69.19) | 72.74 (71.56-73.92) | 27.46 (25.44-29.48) | 45.21 (43.25-47.18) | 75.56 (74.75-76.37) | 39.72 (37.8-41.64) |

**Supplementary Table 2.** AF and dementia-related mortality among older adults stratified by sex and race in the United States from 2000 to 2020.

| **State** | **AAMR (95% CI)** |
| --- | --- |
| Alabama | 41.1 (40.01-42.19) |
| Alaska | 54.09 (49.23-58.96) |
| Arizona | 27.08 (26.32-27.83) |
| Arkansas | 37.18 (35.9-38.46) |
| California | 46.27 (45.85-46.7) |
| Colorado | 61.68 (60.25-63.11) |
| Connecticut | 44.94 (43.78-46.1) |
| Delaware | 43.76 (41.25-46.27) |
| District of Columbia | 26.41 (23.92-28.89) |
| Florida | 25.86 (25.5-26.22) |
| Georgia | 33.33 (32.53-34.13) |
| Hawaii | 31.05 (29.47-32.64) |
| Idaho | 49.9 (47.73-52.06) |
| Illinois | 33.19 (32.61-33.77) |
| Indiana | 50.71 (49.69-51.73) |
| Iowa | 43 (41.79-44.2) |
| Kansas | 32.98 (31.81-34.15) |
| Kentucky | 57.72 (56.34-59.09) |
| Louisiana | 27.94 (26.98-28.9) |
| Maine | 50.11 (48.09-52.13) |
| Maryland | 54.18 (53.03-55.33) |
| Massachusetts | 38.86 (38.04-39.67) |
| Michigan | 37.25 (36.57-37.94) |
| Minnesota | 78.52 (77.18-79.86) |
| Mississippi | 45.67 (44.17-47.17) |
| Missouri | 35.85 (35-36.71) |
| Montana | 47.96 (45.57-50.35) |
| Nebraska | 57.43 (55.52-59.34) |
| Nevada | 20.28 (19.11-21.46) |
| New Hampshire | 56.16 (53.84-58.48) |
| New Jersey | 38.75 (38.02-39.47) |
| New Mexico | 28.76 (27.35-30.18) |
| New York | 30.58 (30.15-31.02) |
| North Carolina | 55.7 (54.77-56.62) |
| North Dakota | 58.34 (55.39-61.29) |
| Ohio | 56.73 (55.96-57.5) |
| Oklahoma | 57.54 (56.09-58.98) |
| Oregon | 82.47 (80.85-84.08) |
| Pennsylvania | 43.94 (43.35-44.54) |
| Rhode Island | 59.99 (57.57-62.41) |
| South Carolina | 62.44 (61.04-63.84) |
| South Dakota | 46.57 (44.11-49.02) |
| Tennessee | 56.54 (55.41-57.67) |
| Texas | 52.08 (51.47-52.7) |
| Utah | 38.2 (36.52-39.89) |
| Vermont | 78.39 (74.54-82.23) |
| Virginia | 41.52 (40.64-42.4) |
| Washington | 69.58 (68.38-70.79) |
| West Virginia | 57.66 (55.75-59.56) |
| Wisconsin | 49.17 (48.16-50.17) |
| Wyoming | 46.21 (42.73-49.69) |

**Supplementary Table 3.** AF and dementia-related mortality among older adults stratified by state in the United States from 2000 to 2020.

| **Census Region** | **Year** | **AAMR (95% CI)** |
| --- | --- | --- |
| Northeast | 2000 | 24.48 (23.37-25.6) |
| Northeast | 2001 | 25.85 (24.72-26.99) |
| Northeast | 2002 | 27.28 (26.12-28.44) |
| Northeast | 2003 | 27.2 (26.06-28.35) |
| Northeast | 2004 | 28.1 (26.94-29.25) |
| Northeast | 2005 | 30.32 (29.13-31.51) |
| Northeast | 2006 | 30.47 (29.29-31.65) |
| Northeast | 2007 | 32.47 (31.26-33.68) |
| Northeast | 2008 | 33.2 (31.99-34.41) |
| Northeast | 2009 | 32.66 (31.47-33.85) |
| Northeast | 2010 | 37.8 (36.52-39.07) |
| Northeast | 2011 | 40.13 (38.82-41.44) |
| Northeast | 2012 | 41.42 (40.11-42.74) |
| Northeast | 2013 | 44.56 (43.2-45.91) |
| Northeast | 2014 | 45.06 (43.7-46.42) |
| Northeast | 2015 | 46.09 (44.73-47.45) |
| Northeast | 2016 | 46.06 (44.7-47.41) |
| Northeast | 2017 | 48.67 (47.3-50.05) |
| Northeast | 2018 | 50.76 (49.36-52.16) |
| Northeast | 2019 | 50.38 (48.99-51.76) |
| Northeast | 2020 | 63.94 (62.38-65.5) |
| Midwest | 2000 | 26.69 (25.59-27.78) |
| Midwest | 2001 | 28.46 (27.34-29.58) |
| Midwest | 2002 | 32.22 (31.03-33.4) |
| Midwest | 2003 | 33.71 (32.5-34.91) |
| Midwest | 2004 | 33.45 (32.25-34.64) |
| Midwest | 2005 | 37.4 (36.15-38.65) |
| Midwest | 2006 | 36.21 (34.99-37.43) |
| Midwest | 2007 | 37.74 (36.5-38.97) |
| Midwest | 2008 | 40.14 (38.88-41.41) |
| Midwest | 2009 | 39.53 (38.28-40.77) |
| Midwest | 2010 | 41.22 (39.96-42.49) |
| Midwest | 2011 | 44.24 (42.94-45.53) |
| Midwest | 2012 | 47.49 (46.16-48.83) |
| Midwest | 2013 | 51.12 (49.74-52.49) |
| Midwest | 2014 | 52.57 (51.19-53.96) |
| Midwest | 2015 | 53.42 (52.03-54.8) |
| Midwest | 2016 | 54.77 (53.37-56.16) |
| Midwest | 2017 | 58.62 (57.18-60.05) |
| Midwest | 2018 | 62.36 (60.9-63.83) |
| Midwest | 2019 | 63.18 (61.71-64.65) |
| Midwest | 2020 | 78.85 (77.21-80.48) |
| South | 2000 | 23.31 (22.44-24.18) |
| South | 2001 | 26.4 (25.48-27.32) |
| South | 2002 | 28.11 (27.17-29.06) |
| South | 2003 | 30.28 (29.3-31.25) |
| South | 2004 | 29.3 (28.35-30.25) |
| South | 2005 | 32.65 (31.66-33.65) |
| South | 2006 | 33.52 (32.53-34.51) |
| South | 2007 | 35.16 (34.16-36.17) |
| South | 2008 | 37.23 (36.21-38.25) |
| South | 2009 | 36.61 (35.61-37.61) |
| South | 2010 | 40.69 (39.65-41.74) |
| South | 2011 | 43.93 (42.87-44.99) |
| South | 2012 | 45.83 (44.76-46.89) |
| South | 2013 | 47.79 (46.71-48.86) |
| South | 2014 | 48.09 (47.02-49.15) |
| South | 2015 | 48.93 (47.87-49.98) |
| South | 2016 | 49.14 (48.1-50.19) |
| South | 2017 | 52.94 (51.87-54.02) |
| South | 2018 | 55.37 (54.29-56.45) |
| South | 2019 | 57.7 (56.6-58.79) |
| South | 2020 | 69.69 (68.51-70.88) |
| West | 2000 | 28.89 (27.6-30.18) |
| West | 2001 | 30.87 (29.56-32.19) |
| West | 2002 | 32.02 (30.69-33.34) |
| West | 2003 | 36.53 (35.13-37.92) |
| West | 2004 | 37.68 (36.27-39.08) |
| West | 2005 | 39.13 (37.73-40.54) |
| West | 2006 | 40.05 (38.65-41.45) |
| West | 2007 | 40.23 (38.85-41.61) |
| West | 2008 | 43.52 (42.11-44.93) |
| West | 2009 | 40.79 (39.44-42.14) |
| West | 2010 | 45.75 (44.34-47.16) |
| West | 2011 | 48.57 (47.14-49.99) |
| West | 2012 | 49.95 (48.53-51.38) |
| West | 2013 | 52.25 (50.81-53.69) |
| West | 2014 | 52.25 (50.83-53.67) |
| West | 2015 | 54.15 (52.72-55.57) |
| West | 2016 | 56.18 (54.74-57.61) |
| West | 2017 | 58.68 (57.22-60.13) |
| West | 2018 | 58.44 (57.01-59.87) |
| West | 2019 | 60.63 (59.19-62.07) |
| West | 2020 | 68.4 (66.89-69.92) |

**Supplementary Table 4**. AF and dementia-related mortality among older adults stratified by region in the United States from 2000 to 2020.

| **Year** | **Metropolitan** | **Nonmetropolitan** |
| --- | --- | --- |
| 2000 | 25.52 (24.93-26.11) | 25.3 (24.11-26.49) |
| 2001 | 27.69 (27.08-28.31) | 27.74 (26.5-28.99) |
| 2002 | 29.61 (28.98-30.23) | 30.11 (28.81-31.4) |
| 2003 | 31.29 (30.65-31.93) | 33.44 (32.08-34.8) |
| 2004 | 31.08 (30.45-31.71) | 34.78 (33.4-36.16) |
| 2005 | 34.11 (33.46-34.76) | 37.04 (35.62-38.46) |
| 2006 | 34.54 (33.89-35.19) | 36.35 (34.95-37.74) |
| 2007 | 35.96 (35.3-36.61) | 37.55 (36.14-38.96) |
| 2008 | 37.62 (36.96-38.28) | 41.62 (40.14-43.1) |
| 2009 | 36.61 (35.96-37.25) | 40.73 (39.27-42.18) |
| 2010 | 40.98 (40.31-41.66) | 42.8 (41.33-44.28) |
| 2011 | 43.96 (43.27-44.65) | 45.32 (43.82-46.83) |
| 2012 | 45.45 (44.76-46.14) | 49.84 (48.27-51.4) |
| 2013 | 48.12 (47.42-48.82) | 52.67 (51.07-54.28) |
| 2014 | 48.3 (47.61-49) | 54.75 (53.12-56.37) |
| 2015 | 49.46 (48.77-50.16) | 55.92 (54.29-57.55) |
| 2016 | 50.27 (49.58-50.97) | 56.77 (55.14-58.4) |
| 2017 | 52.82 (52.12-53.53) | 63.51 (61.8-65.22) |
| 2018 | 54.74 (54.03-55.45) | 66.57 (64.83-68.31) |
| 2019 | 56.01 (55.3-56.72) | 68.93 (67.17-70.69) |
| 2020 | 67.64 (66.87-68.42) | 83.96 (82.03-85.89) |

**Supplementary Table 5.** AF and dementia-related mortality among older adults stratified by urbanization in the United States from 2000 to 2020.

**
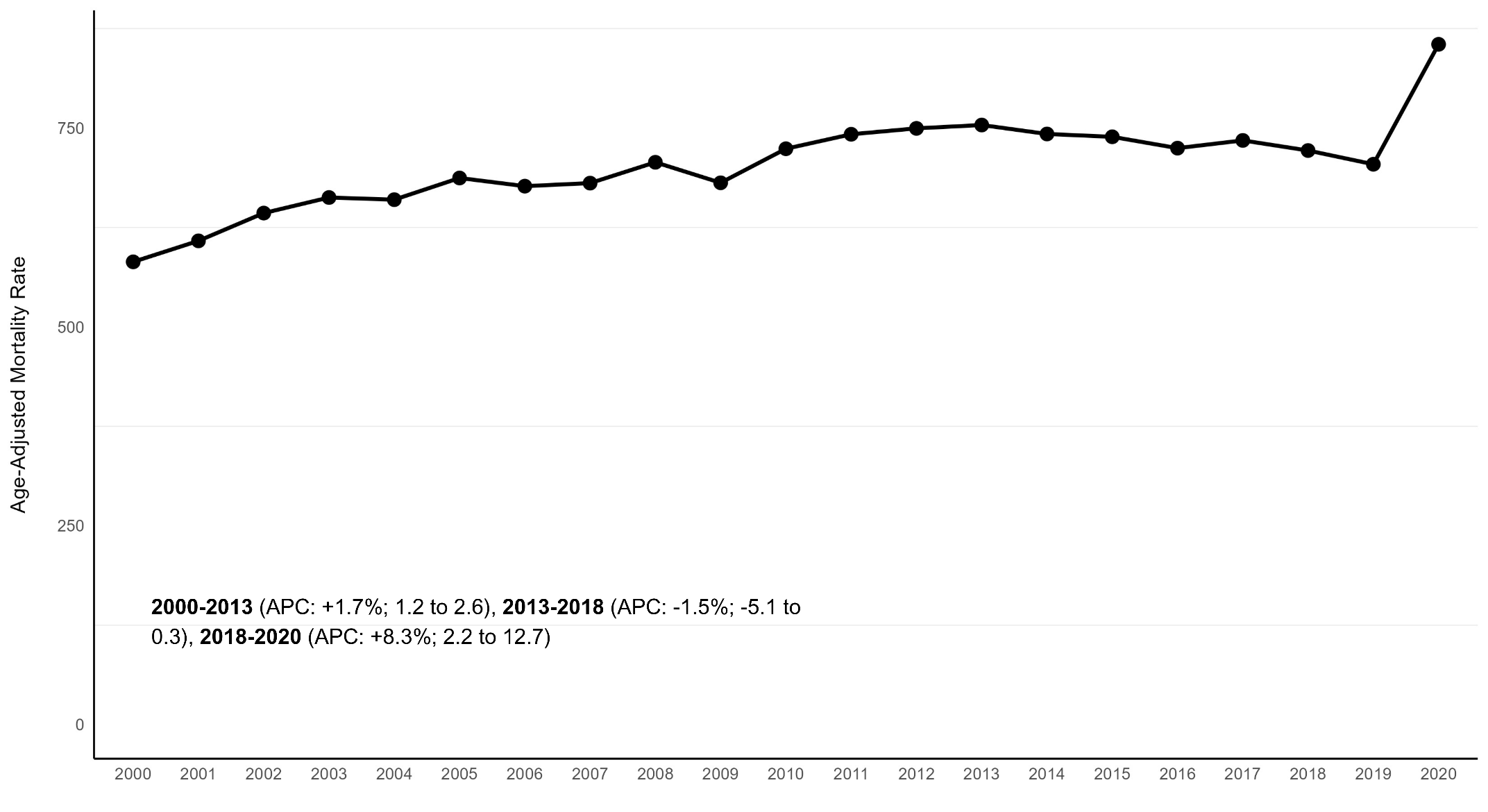
**

**Supplementary Figure 1.** Trends in dementia-related mortality in the United States among older adults from 2000 to 2020.

**
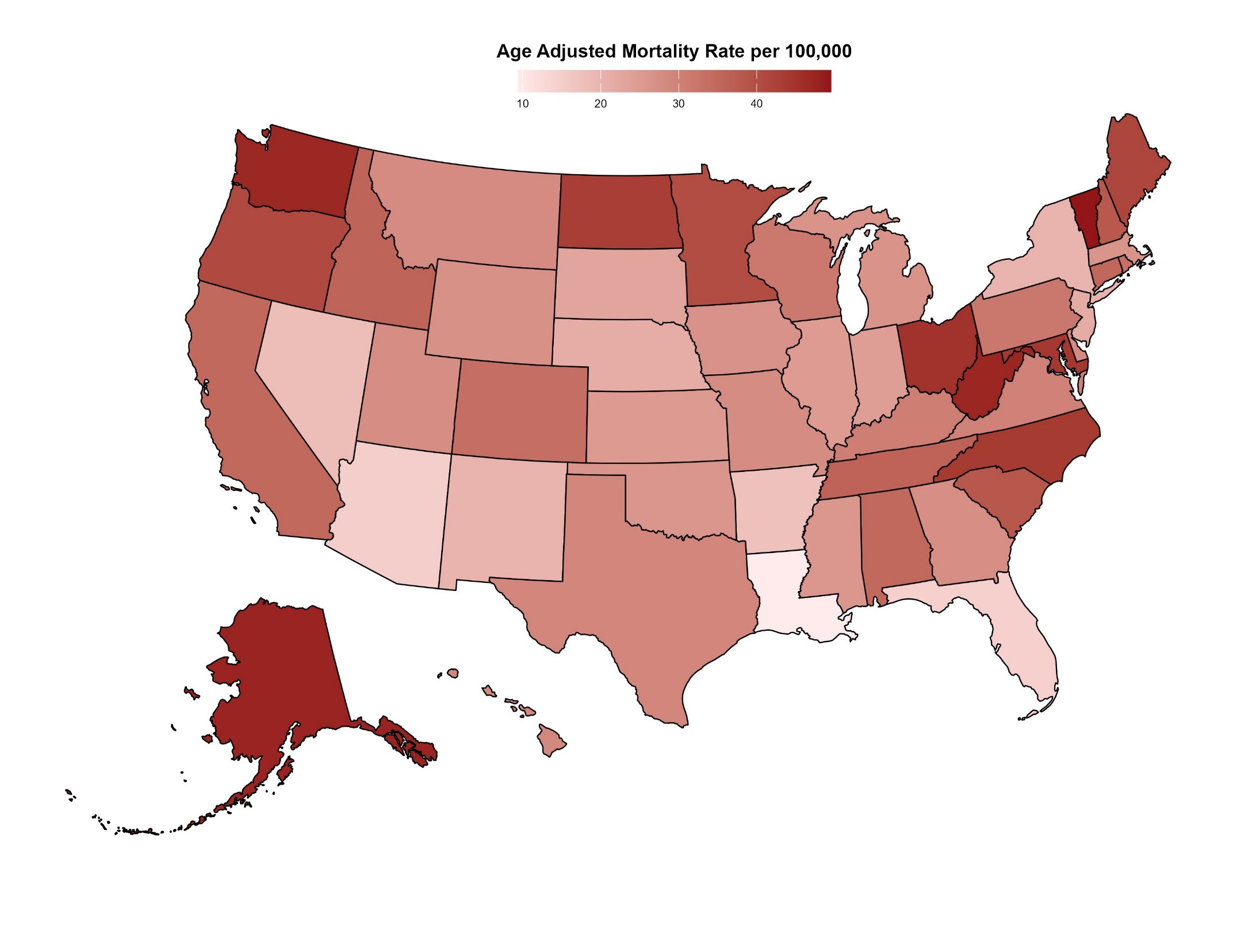
**

**Supplementary Figure 2.** Trends in AF and dementia related mortality in the United States among older adults from 2000 to 2004.


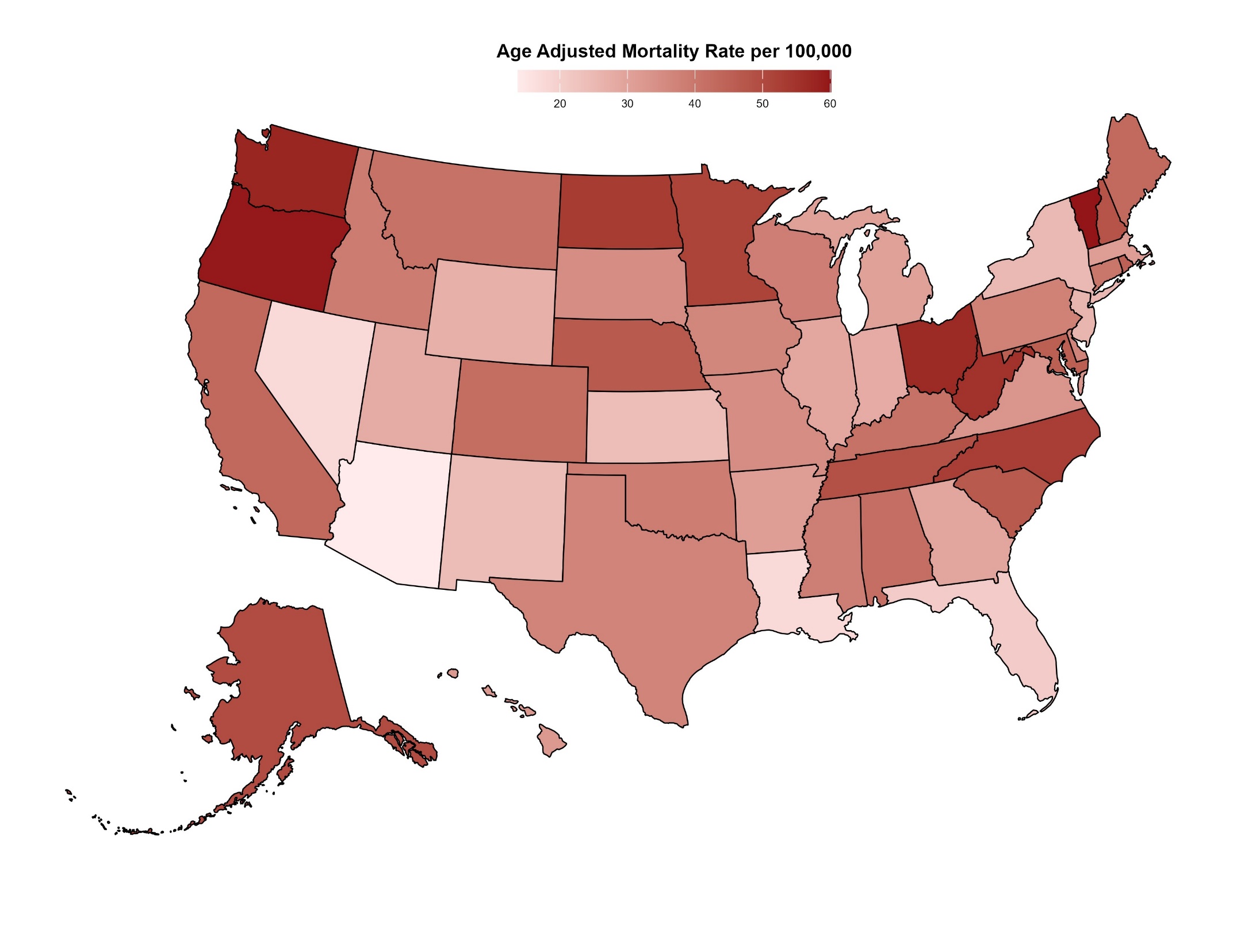


**Supplementary Figure 3.** Trends in AF and dementia related mortality in the United States among older adults from 2005 to 2009.


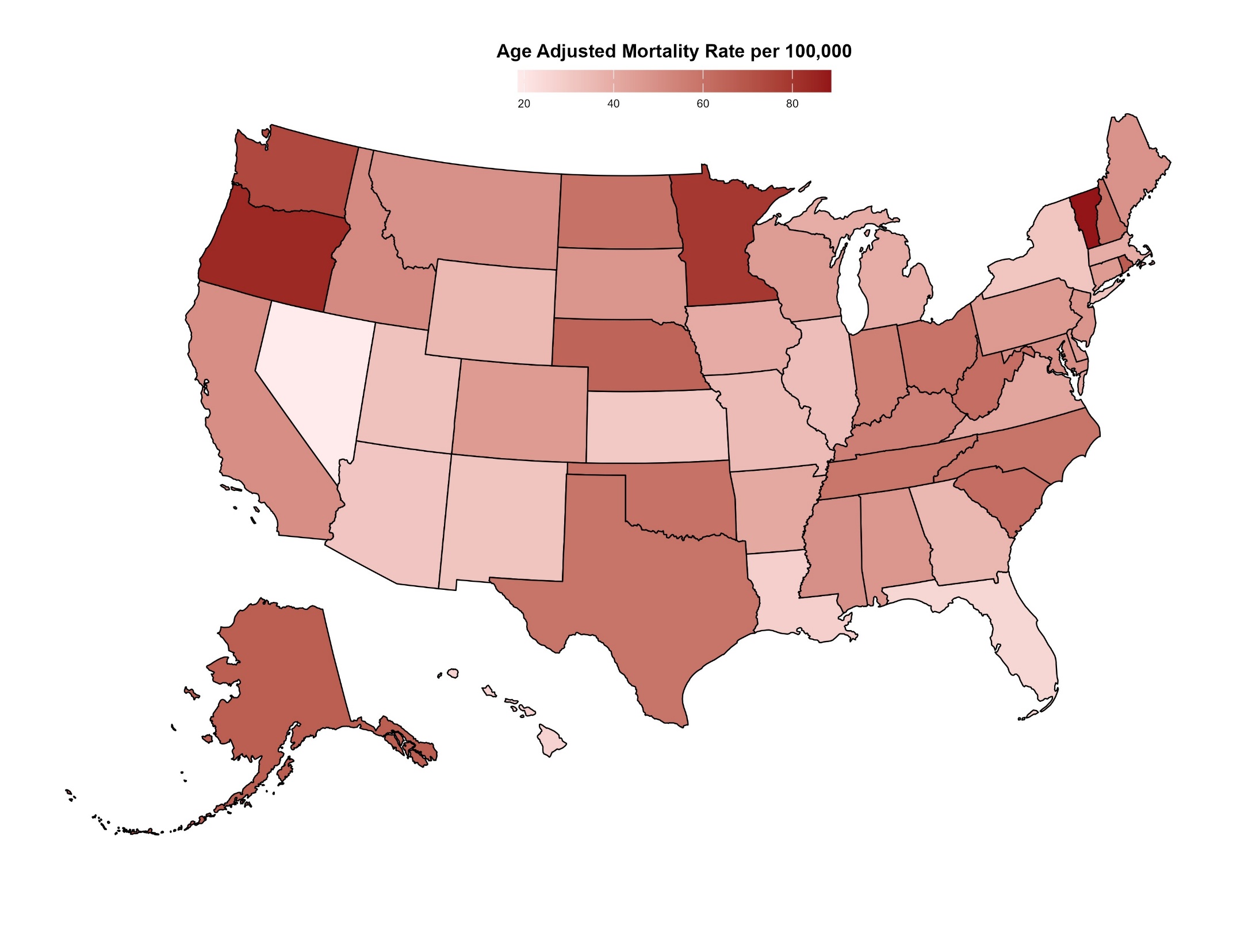


**Supplementary Figure 4.** Trends in AF and dementia related mortality in the United States among older adults from 2010 to 2014.


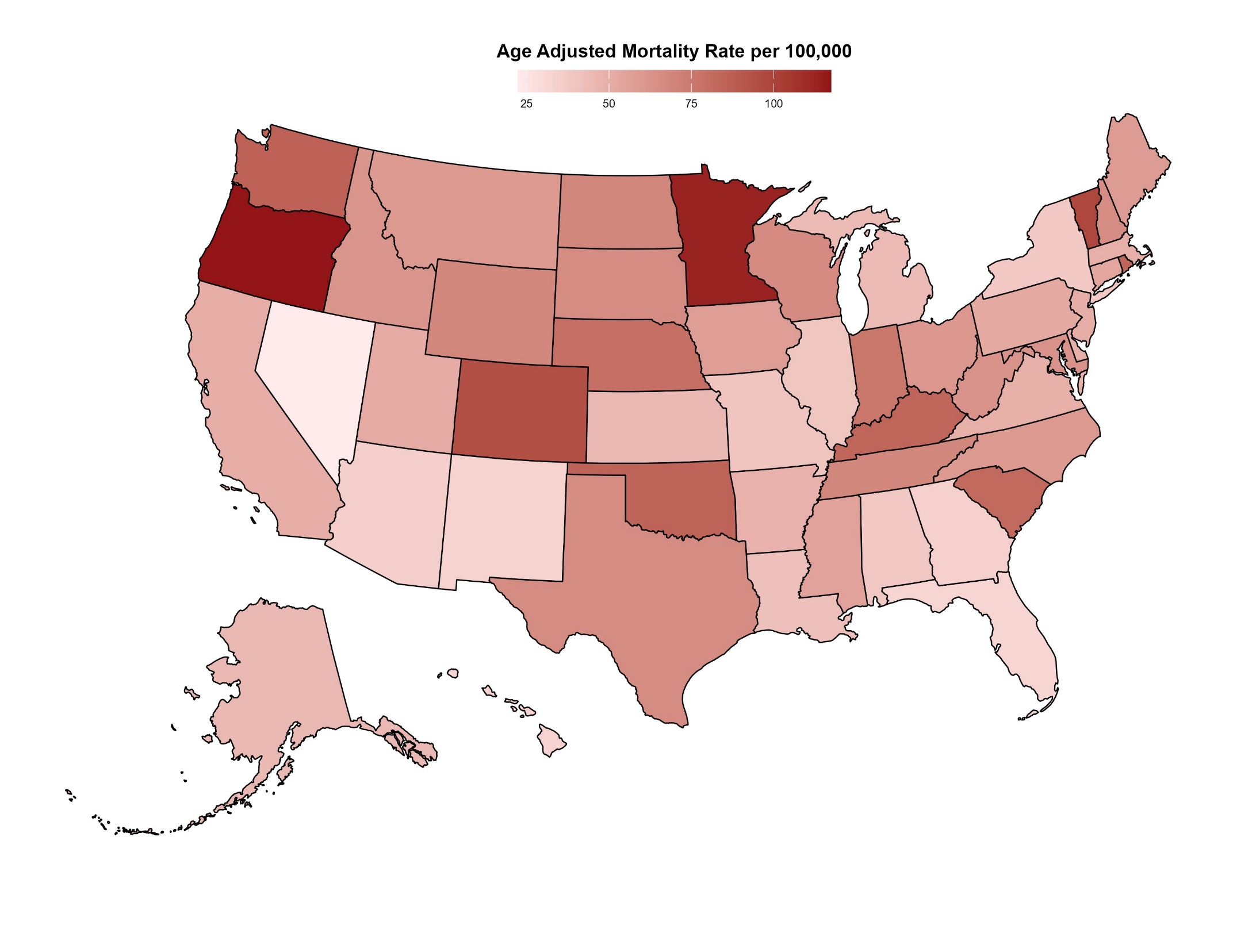


**Supplemental Figure 5.** Trends in AF and dementia related mortality in the United States among older adults from 2015 to 2019.


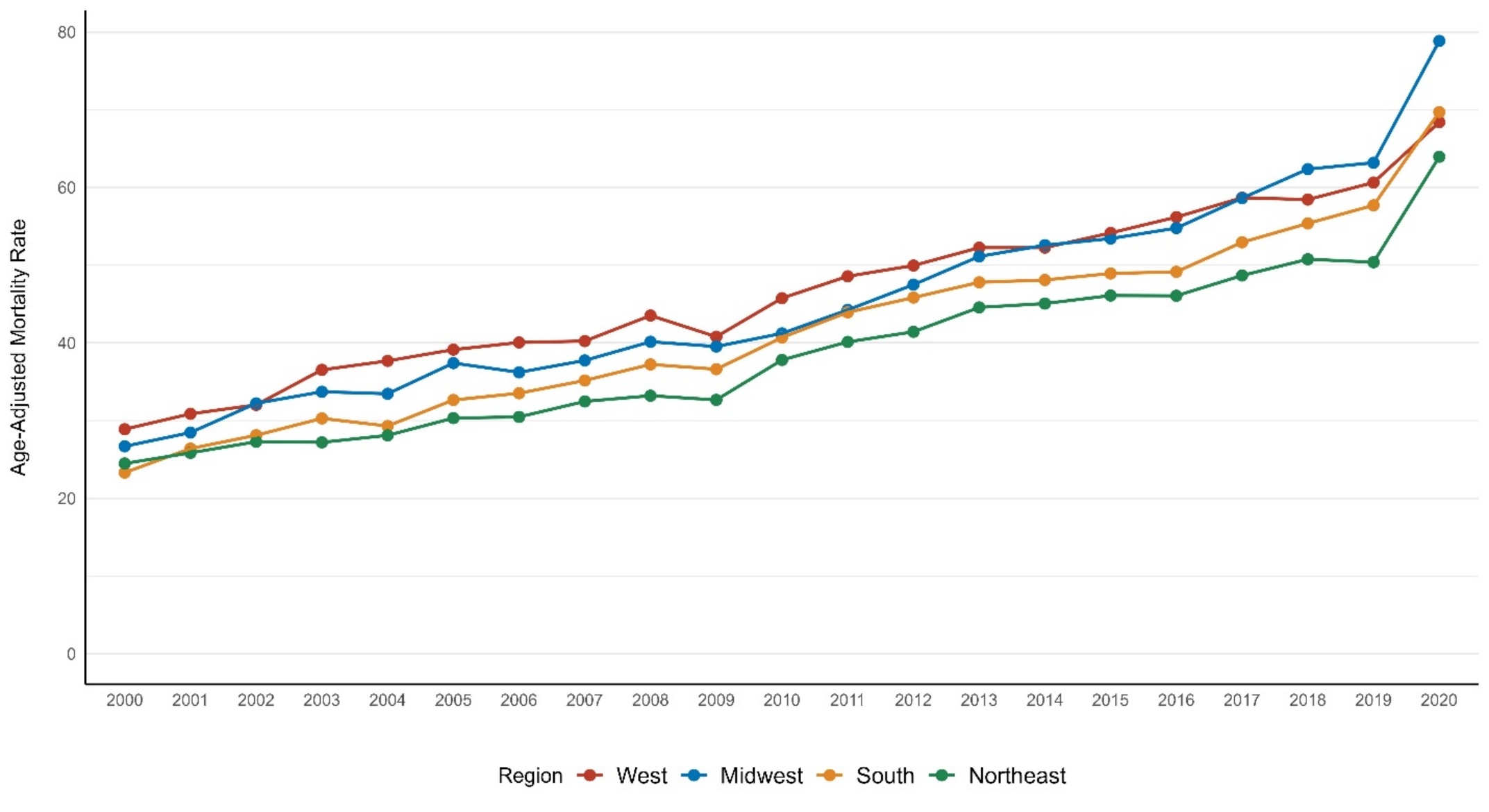

 **Supplementary Figure 6.** Trends in AF and dementia-related mortality among older adults stratified by region in the United States from 2000 to 2020.
